# Supplementary material for: Exploring Response to Immunotherapy in Non-Small Cell Lung Cancer Using Delta-Radiomics
Source: Cancers (Basel). 2022 Jan 11;14(2):350. doi: 10.3390/cancers14020350 (PMC8773717; doi:10.3390/cancers14020350)
Supplement: Supplementary file 1 [file cancers-14-00350-s001.zip › cancers-1494217-supplementary.pdf]

Supplementary Materials

Table S1. Delta-radiomic features calculated with the Absolute difference method that resulted statistically different in the 3 response classes. The results are presented as median values for each class and p-values.

Absolute Difference					
**Class**	**Name**	**PD**	**PR**	**SD**	**p-Value**
firstorder	TotalEnergy	$-6{,}060 \times 10^9$	$1{,}046 \times 10^{11}$	$-1{,}200 \times 10^7$	0.0212
gldm	DependenceNonUniformity	−37,24	676,5	−39,31	0.0415
gldm	GrayLevelNonUniformity	−341,5	2328	−63,22	0.0277
glcm	Contrast	0,1798	−0,3042	0,2766	0.0326
glrlm	GrayLevelNonUniformity	−122,3	1175	−55,45	0.0387
glrlm	RunLengthNonUniformity	−509,3	3777	−250,3	0.0329
glszm	ZoneVariance	−3847	34668	−794,1	0.0237
glszm	LargeAreaEmphasis	−3835	34908	−846,4	0.0234
glszm	LargeAreaLowGrayLevelEmphasis	−72,54	159,0	−1,676	0.0165
glszm	LargeAreaHighGrayLevelEmphasis	−17371	$1{,}496 \times 10^7$	−59729	0,0276

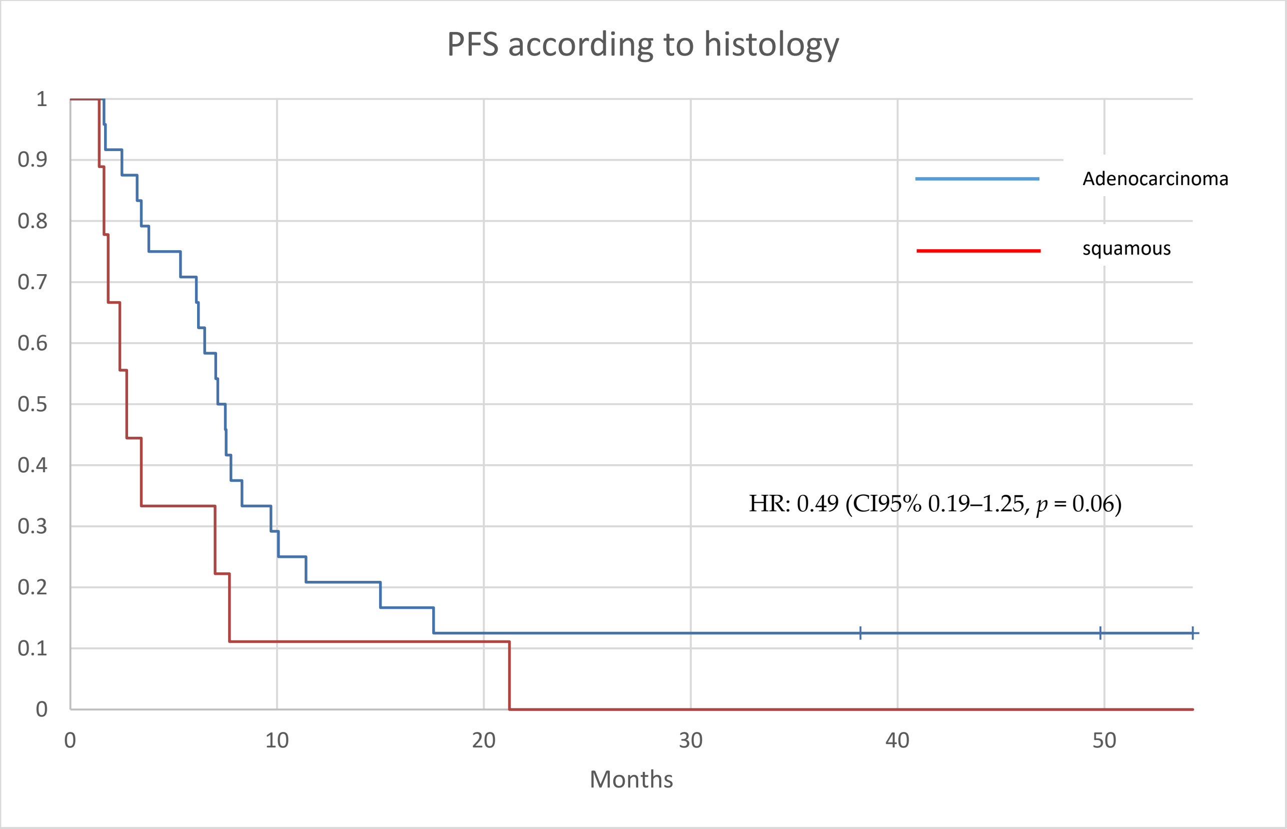

Figure S1. Univariate analysis of progression free survival (PFS) according to histology.

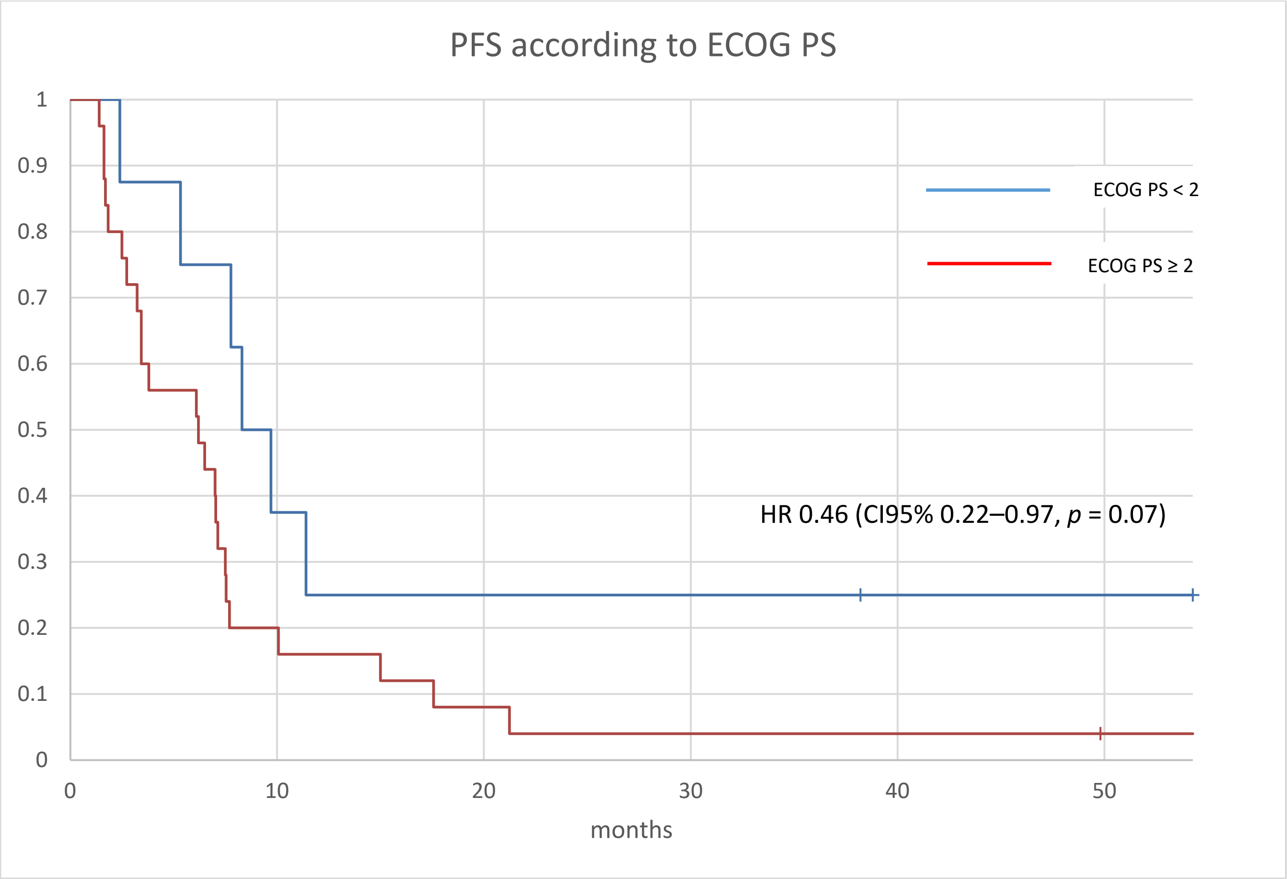

Figure S2. univariate analysis of progression free survival according to the eastern cooperative oncology group (ECOG PS).

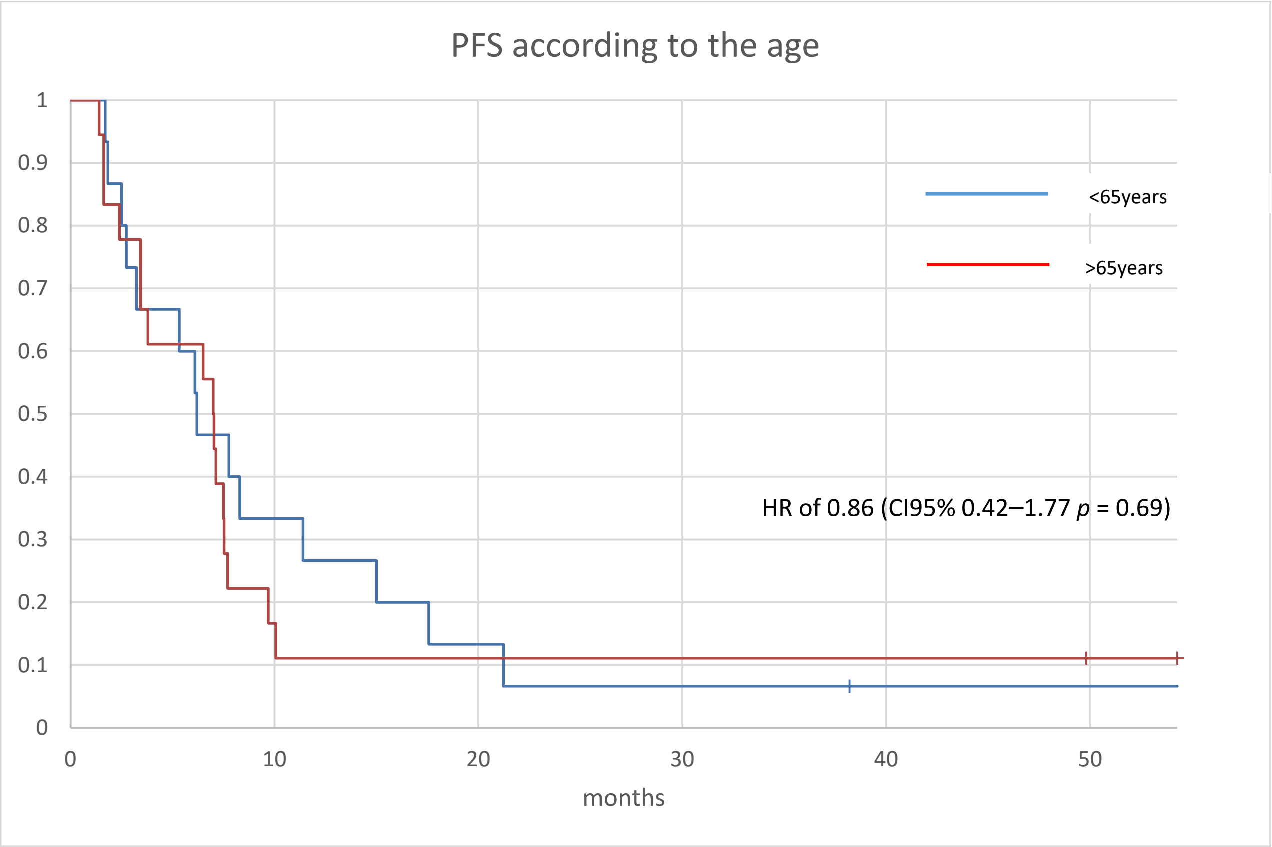

Figure S3. univariate analysis of progression free survival (PFS) according to the age.
